# Melatonin and cancer: towards a time-sensitive oncological adjunct

**DOI:** 10.1016/j.clinsp.2025.100834

**Published:** 2025-11-18

**Authors:** Ricardo Hsieh, Silvia Vanessa Lourenço, Edmund Chada Baracat, José Cipolla-Neto, José Maria Soares Junior

**Affiliations:** a Laboratório de Investigação em Ginecologia Estrutural e Molecular (LIM-58), Disciplina de Ginecologia, Departamento de Obstetrícia e Ginecologia, Hospital das Clínicas, Faculdade de Medicina, Universidade de São Paulo, São Paulo, SP, Brazil; b Department of Surgery, Hospital das Clínicas, Faculdade de Medicina, Universidade de São Paulo, São Paulo, SP, Brazil; c ICB, Departamento de Fisiologia, Laboratório de Neurobiologia, Universidade de São Paulo, São Paulo, SP, Brazil; d Laboratório de Imunopatologia da Esquistossomose e outras Parasitoses (LIM-06), Instituto de Medicina Tropical de São Paulo, Faculdade de Medicina da Universidade de São Paulo, São Paulo, SP, Brazil

The intersection between circadian biology and oncology has become increasingly relevant as evidence continues to demonstrate that disruptions in biological rhythms profoundly influence tumorigenesis, cancer progression, and therapeutic response. Among the key molecular pathways linking circadian regulation to cancer biology, melatonin has emerged as a molecule of both mechanistic and therapeutic interest. Traditionally recognized as the principal hormone regulating sleep-wake cycles, melatonin is now considered a pleiotropic agent with antioxidant, anti-proliferative, immunomodulatory, and pro-apoptotic properties. Its potential as an adjuvant in cancer management is generating renewed attention and debate within the oncological community.

Circadian disruption has been recognized as a carcinogenic factor, influencing cellular metabolism, genomic stability, and the tumor microenvironment. Recent studies indicate that circadian dysregulation not only predisposes individuals to malignancy but also compromises therapeutic efficacy and survivorship outcomes [1]. The clinical implications of this are profound, as timing of therapy or chronotherapy has been proposed as a strategy to enhance treatment efficacy while minimizing toxicity. Thus, melatonin, as the primary endocrine signal of the circadian clock, occupies a central position in this present discussion.

Cancer-related fatigue remains one of the most disabling symptoms for patients across tumor types and treatment modalities. The search for non-toxic, accessible, and effective interventions has led to the investigation of melatonin supplementation in randomized clinical trials. A recent double-blind placebo-controlled trial in women with early-stage breast cancer undergoing radiotherapy found that melatonin did not significantly reduce fatigue, yet secondary outcomes suggested benefits in sleep quality and global well-being [2]. In contrast, a meta-analysis pooling randomized controlled trials reported that melatonin supplementation was associated with reductions in fatigue intensity, although heterogeneity across studies must be acknowledged [3].

Additionally, more encouraging results have been documented in the setting of chemotherapy. In a randomized trial, melatonin supplementation significantly reduced cancer-related fatigue among women receiving cytotoxic treatment for breast cancer, with improved patient-reported outcomes in quality-of-life domains [4]. Long-term follow-up trials also suggest persistent benefits in fatigue reduction without major safety concerns, underscoring melatonin’s favorable risk-benefit profile [5]. Beyond fatigue, melatonin’s impact on broader quality of life indices has been highlighted in a systematic review and meta-analysis, where improvements in sleep, depressive symptoms, and overall symptom burden were observed across heterogeneous cancer populations [6].

Nevertheless, the role of environmental light exposure, particularly Artificial Light at Night (ALAN), adds further complexity to the interface of circadian biology and cancer. Epidemiological evidence suggests that ALAN disrupts melatonin secretion and is associated with increased risk of several cancers, most notably breast and prostate cancer. A recent systematic review and meta-analysis integrating indoor and outdoor ALAN exposures confirmed this association, while also emphasizing methodological challenges in exposure assessment [7]. These findings are aligned with broader literature on circadian disruption among cancer survivors, who frequently experience sleep disturbances, hormonal dysregulation, and reduced quality of life, thereby perpetuating the cycle of vulnerability to poor outcomes [8].

The implications of these findings extend beyond symptom management, touching on cancer prevention, prognosis, and survivorship care. Preclinical data have long demonstrated that melatonin exerts anti-tumor effects through multiple pathways, including inhibition of angiogenesis, modulation of estrogen receptor signaling, and promotion of apoptosis. Translational studies now suggest that these effects may be potentiated when therapy is aligned with circadian timing, reinforcing the concept of chronotherapy [9]. This paradigm shift underscores the need to reimagine oncological care not merely as a pharmacological intervention but as a time-sensitive process informed by circadian principles.

Moreover, the therapeutic potential of melatonin is further supported by interventional clinical data in surgical oncology. In the AMPLCaRe trial, adjuvant melatonin administration following lung cancer resection was associated with signals of reduced recurrence and mortality, though definitive conclusions await further validation [10]. Such evidence underscores the possibility that melatonin, long regarded as a benign over-the-counter supplement, may in fact hold the key to novel adjuvant strategies in oncology.

According to the literature, current evidence suggests that melatonin represents more than a sleep-regulating hormone, and it is a biologically active compound at the crossroads of circadian biology and oncology. While results across clinical trials are mixed, meta-analyses and ongoing research point to clinically meaningful benefits in fatigue, quality of life, and potentially survival. Importantly, melatonin’s safety, affordability, and accessibility position and a unique candidate for incorporation into integrative cancer care models. The growing recognition of circadian disruption as a hallmark of modern oncological risk further strengthens the case for melatonin as both a preventive and therapeutic agent.

In this scenario, the challenge for the next decade is twofold. Firstly, to refine clinical trial designs to capture not only efficacy signals but also the time-of-day dependent effects of melatonin administration. Second, to integrate circadian assessments into oncology practice, thereby tailoring interventions such as melatonin to individual circadian phenotypes. The convergence of chronobiology and oncology offers a unique window of opportunity, one in which melatonin stands out as a promising and time-sensitive oncological adjunct.

## Data availability statement

The datasets generated and/or analyzed during the current study are available from the corresponding author upon reasonable request.

## Conflicts of interest

The authors declare no conflicts of interest.
